# Management of localized gingival recession by two-stage surgical procedure - Double pedicle flap with CTG and coronally advanced flap: A novel technique

**DOI:** 10.4103/0972-124X.51895

**Published:** 2009

**Authors:** T. Prasanth

**Affiliations:** *Graded Specialist (Periodontology) ,01 Air Force Dental Centre, Air Force Station, Palam, Delhi Cantt – 11 0010, India*

**Keywords:** Connective tissue graft, coronally advanced flap, double pedicle flap, gingival recession

## Abstract

Cosmetic treatments have become an integral part of periodontal treatment. One of the commonly used esthetic periodontal procedures is coverage of denuded root surface. While considering the elimination of these defects two criteria should be considered, the esthetic aspects and the functional aspects. This case report has describes a two stage surgical technique using double pedicle flap with connective tissue graft followed by coronally advanced flap for the treatment of a severe localized gingival recession measuring 15 mm. The recession measurement at the end of 12 months was 1 mm. It showed a predictable result at the end of one year. The advantages of this technique are excellent colour matching, dual blood supply to graft and very predictable results. The promising result suggest that this technique can be used in severe gingival recession cases with minimum amount of keratinized tissue.

## INTRODUCTION

Cosmetic treatments have become an integral part of periodontal treatment. One of the commonly used esthetic periodontal procedures is coverage of denuded root surface. Gingival recession is the apical shift of the marginal gingiva from its normal position on the crown of the tooth to levels on the root surface beyond the cemento enamel junction.[1] While considering the elimination of these defects, two criteria should be considered are the esthetic and the functional aspects. The former being the main concern of patient and the later of clinician and hence all attempt should be made to consider both while doing root coverage procedure.[2] This new technique describes a two stage surgical procedure to manage localized gingival recession case. The first stage compris of double pedicle flap along with connective tissue graft and the second stage consist of coronally advanced flap after a period of three months of the first surgery.

## CASE REPORT

A healthy 35-year-old serving soldier reported to the department of dental surgery, Armed Forces Medical College, Pune, India with chief complaints of receding gums, pus discharge along with unpleasant look in relation to the left upper tooth. Dental history revealed that he had visited various service hospitals for treatment, but no surgical treatment was received. Recently he was advised extraction of the same tooth.

**Figure 1:**
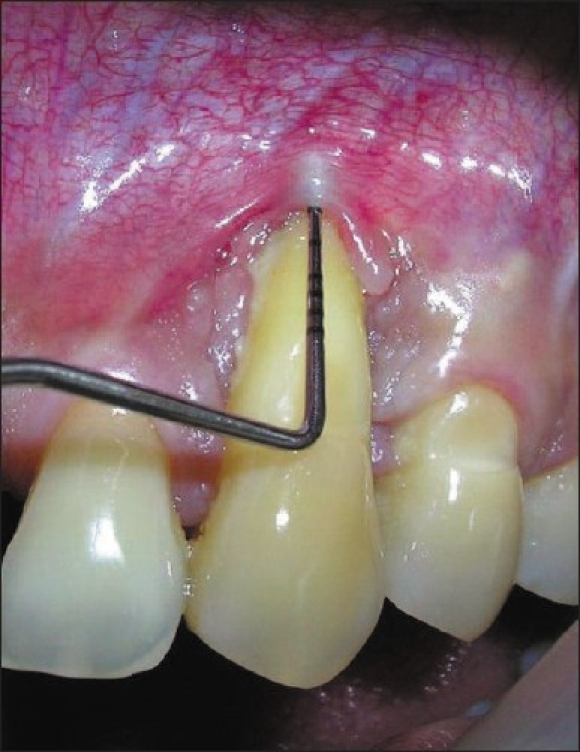
Pre operative (CAL-17 mm)

Pre surgical examination showed Millers Class III gingival recession present on left maxillary canine. The recession was measuring 15 mm and probing depth was 02 mm causing a 17 mm clinical attachment loss [Figure 1]. The tooth was having grade II mobility. Pus discharge was present on digital pressure. He was also wearing acrylic crowns on both central incisors.

Vitality test was carried out on left maxillary canine and the tooth was non vital. Phase I therapy was performed which consist of thorough scaling and root planing followed by endodontic treatment on the left maxillary canine. The tooth was splinted to adjacent premolar with intracoronal wire and resin restorative material. The case was evaluated periodically for a period of 03 months. The case was taken up initially for gingival recession coverage using double pedicle flap with connective tissue graft. The patient was explained about the procedure and a written consent obtained.

### Surgical technique

#### Stage 1- Double pedicle flap with connective tissue graft

**Figure 2:**
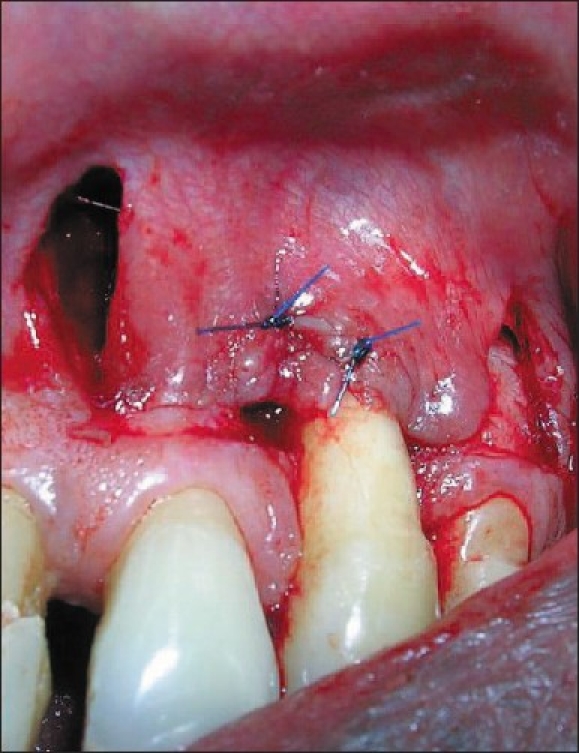
Double pedicle flap sutured

After giving local anesthesia, the exposed root surface was thoroughly planed to remove any plaque, calculus and soft root structure. Two horizontal incisions were given on both mesial and distal sides of defect 01 mm away from the gingival margin of the adjacent tooth. Then two vertical incisions were made perpendicular to the initial incisions on either side which were extending well into the alveolar mucosa. Partial thickness pedicles were reflected on either side of the canine. The reflection was carried out to a level that would permit free movements of the mesial and distal pedicle flaps. Both these pedicles were rotated over the defect to make sure they would remain over the defect without any tension. Then both pedicles were suture with 6-0 polypropylene suture [Figure 2].

**Figure 3:**
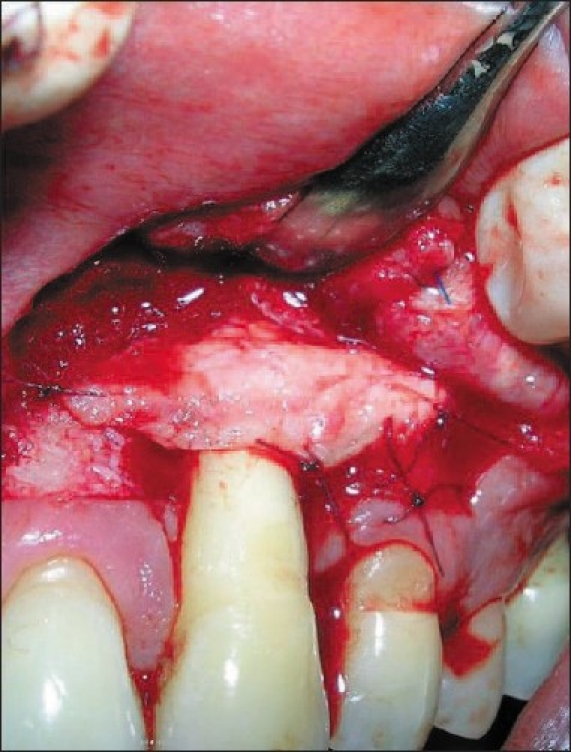
CTG placement

A connective tissue graft was then harvested from the palate. The graft was then transferred to the recipient site with adequate extension to the sound bone and stabilized with 6-0 vicryl suture [Figure 3]. The pedicles were repositioned over the stabilized graft and sutures with 6-0 polypropylene suture. No attempt was made to cover the defect fully. Light pressure was applied to the grafted area with wet gauze and the periodontal dressing was given.

Patient was placed on 0.12% chorhexidine mouth wash for 04 weeks. Cap Amoxicillin 500 mg 03 times and Tab Ibuprofen 400 mg 03 times were prescribed for 05 days. Suture was removed 01 week, post-operatively. The patient was reviewed regularly for a period of 03 months.

**Figure 4:**
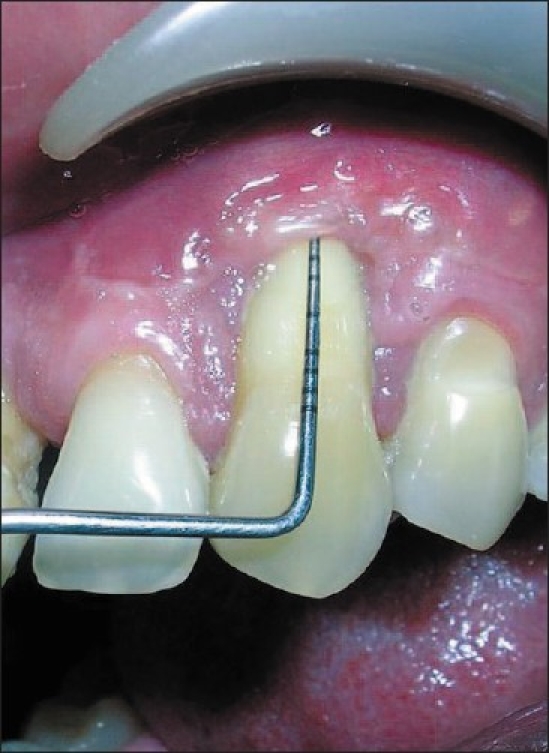
3 month Post op after stage 1 (CAL-8 mm)

At the end of three months, the recession was reduced to 07 mm, probing depth was 01 mm and clinical attachment loss was 08 mm [Figure 4]. The width of keratinized gingiva was increased to 03 mm which was absent pre-surgically. The case was taken up for second stage of surgery and a coronally advanced flap was planned.

#### Stage 2- Coronally advanced flap

**Figure 5:**
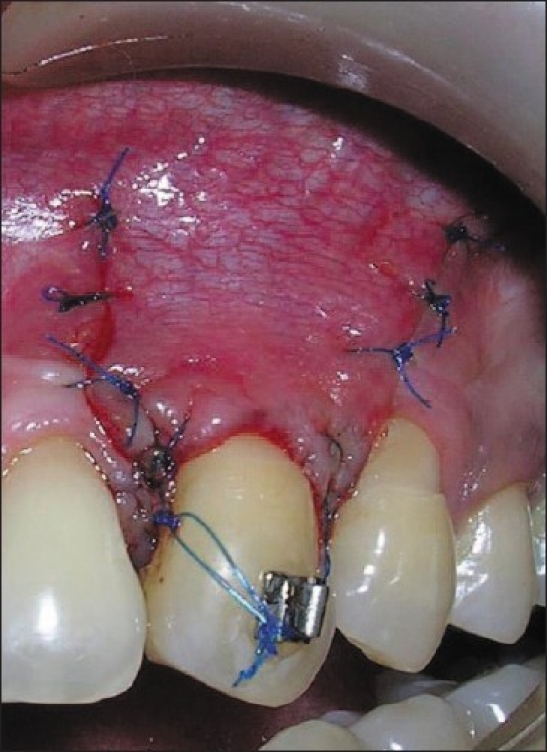
Stage 2 - Coronally advanced flap

**Figure 6:**
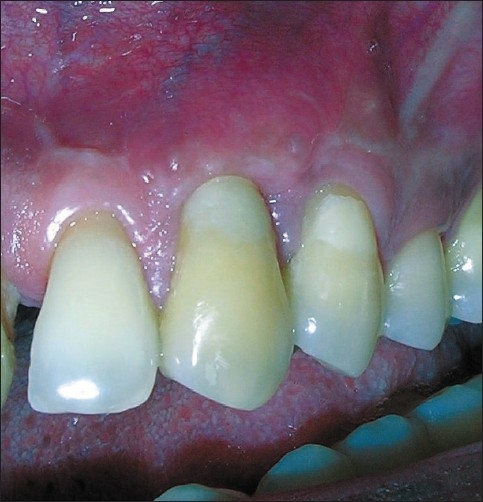
Post op 12 months

**Figure 7:**
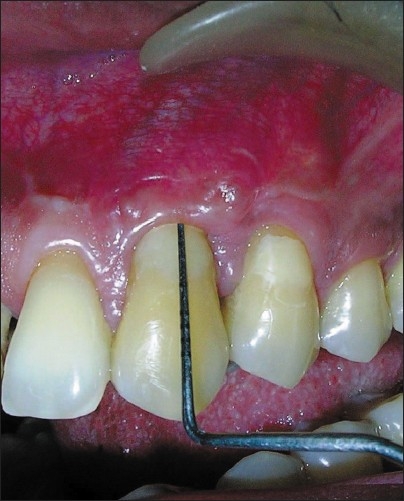
Post op 12 months (CAL - 2 mm)

A partial thickness flap was raised around the defect with the help of two horizontal and two vertical incisions on either sides of defect without involving the marginal gingiva of adjacent teeth. To facilitate a tension free coronal displacement, its base was separated from the periosteum with the help of a periosteal releasing incision. The flap was advanced coronally and sutured at the level of CEJ using 5-0 polypropylene suture. First, an anchor suture was placed around the tooth and additional support was taken from an orthodontic bracket which was fixed to the crown prior to the surgery. The flap was secured with two interproximal sutures and lateral sutures [Figure 5]. Periodontal dressing was given. Necessary post op care instructions and medications were given. The suture was removed after 01week and the patient was recalled regularly for a period of 12 months. The recession measurement at the end of 12 months was 1 mm, PD was 1mm and CAL was 2mm [Figures 6 and 7].

## DISCUSSION

In 1956, Grupe and Warren made initial attempt to cover the denuded root using pedicle flap.[3] Later, many workers contributed further by modifying the lateral sliding flap technique. Cohen and Ross[4] used double papilla repositioned flap in gingival recession coverage. The benefits of choosing this technique was minimal exposure of the underlying periosteum at the interdental donor sites, rapid wound healing at the donor site. The main indication of this technique is the presence of wide recession with adequate door tissue on either side.

Due to unsatisfactory result with sliding flaps and a frequent lack of donor material associated with multiple gingival recessions, a two-stage surgical procedure was advocated by various workers. Bernimoulin[5] and his co worker in 1975 described a two-stage technique for gingival recession coverage. Here they initially placed a free gingival graft apical to the recession to increase the width of attached gingiva. Two months later, a full thickness muco periosteal flap was raised and advanced coronally to the desired position to get gingival recession coverage. They reported clinically significant increase in the width of keratinized gingiva along with gingival recession coverage.

Later, various authors described surgical procedures which utilized the similar procedure described by Bernimoulin and co worker. Maynard in 1977, Caffesse and Guinard and Tenenbaum in 1980 utilized coronally repositioned flap after free gingival graft with satisfactory results. Later on pedicle periodontal flap was introduced for the treatment of gingival recession coverage in combination with connective tissue graft. The rationale for the use of this technique was the presence of insufficient attached gingiva in the donor site. Harris[6] reported the use of connective tissue graft and partial thickness double pedicle graft for obtaining root coverage in 1992. Mean root coverage of 97.2 % was obtained with 100% coverage in 24 of the 30 sites.

The main advantages of this technique are excellent colour matching and good tissue contour to the treated site. It produces a very predictable result because of the dual blood supply to the connective tissue graft. The factors which could affect the success of this procedure include size of pedicles, less tension of the pedicles, adequate vascular nourishment to the graft gingival graft and plaque control during the post operative period. Another advantage of this procedure is that it can be used predictably in cases where minimum or absent keratinized tissue at the affected site.

The use of connective tissue with double pedicle graft is a very effective method to obtain root coverage for severe localized gingival recession cases. The initial surgery resulted in increase the width of attached gingiva. The tissue that covers the root surface seems to attach tightly throughout the follow up period. There were no signs of inflammation in the operated sites. It is still unclear what type of attachment occurs after the root coverage procedure. Histological study of similar kind of procedure in the past showed that the healing may be due to repair but not by regeneration.[7] The second stage of surgery performed three months after the first stage. The width of attached gingiva was adequate to perform this. The advantages of coronally advanced flap are a simple procedure and there is no requirement of a second surgical site.[8]

## CONCLUSION

This new technique has shown that surgical technique using connective tissue graft and double pedicle flap followed by coronally advanced flap in the treatment of root coverage can give a very predictable result. The advantages of this technique are excellent colour matching, dual blood supply to graft and very predictable results. The factors which could affect the success of this technique are size of pedicles, previous CTG technique and plaque control during the post op period. This technique can be used in sever gingival recession cases with minimum amount of keratinized tissue Clinical studies using large sample size and longer duration are advised to determine the success and predictability of this technique.
